# Cation non-stoichiometry in Fe:SrTiO_3_ thin films and its effect on the electrical conductivity†

**DOI:** 10.1039/d1na00358e

**Published:** 2021-09-10

**Authors:** Maximilian Morgenbesser, Stefanie Taibl, Markus Kubicek, Alexander Schmid, Alexander Viernstein, Niklas Bodenmüller, Christopher Herzig, Federico Baiutti, Juan de Dios Sirvent, Maciej Oskar Liedke, Maik Butterling, Andreas Wagner, Werner Artner, Andreas Limbeck, Albert Tarancon, Jürgen Fleig

**Affiliations:** Institute of Chemical Technologies and Analytics, TU Wien Getreidemarkt 9-164/EC 1060 Vienna Austria markus.kubicek@tuwien.ac.at; Catalonia Institute for Energy Research (IREC) Jardins de Les Dones de Negre 1, 08930 Sant Adria del Besos Barcelona Spain; Institute of Radiation Physics, Helmholtz-Zentrum Dresden-Rossendorf (HZDR) Bautzner Landstraße 400 01328 Dresden Germany; Fachbereich Röntgenzentrum, TU Wien Getreidemarkt 9 1060 Vienna Austria; ICREA 23 Passeig Lluis Companys Barcelona 08010 Spain

## Abstract

The interplay of structure, composition and electrical conductivity was investigated for Fe-doped SrTiO_3_ thin films prepared by pulsed laser deposition. Structural information was obtained by reciprocal space mapping while solution-based inductively-coupled plasma optical emission spectroscopy and positron annihilation lifetime spectroscopy were employed to reveal the cation composition and the predominant point defects of the thin films, respectively. A severe cation non-stoichiometry with Sr vacancies was found in films deposited from stoichiometric targets. The across plane electrical conductivity of such epitaxial films was studied in the temperature range of 250–720 °C by impedance spectroscopy. This revealed a pseudo-intrinsic electronic conductivity despite the substantial Fe acceptor doping, *i.e.* conductivities being several orders of magnitude lower than expected. Variation of PLD deposition parameters causes some changes of the cation stoichiometry, but the films still have conductivities much lower than expected. Targets with significant Sr excess (in the range of several percent) were employed to improve the cation stoichiometry in the films. The use of 7% Sr-excess targets resulted in near-stoichiometric films with conductivities close to the stoichiometric bulk counterpart. The measurements show that a fine-tuning of the film stoichiometry is required in order to obtain acceptor doped SrTiO_3_ thin films with bulk-like properties. One can conclude that, although reciprocal space maps give a first hint whether or not cation non-stoichiometry is present, conductivity measurements are more appropriate for assessing SrTiO_3_ film quality in terms of cation stoichiometry.

## Abstract 2

The interplay of structure, composition and electrical conductivity was investigated for Fe-doped SrTiO_3_ thin films prepared by pulsed laser deposition.

## Graphical abstract

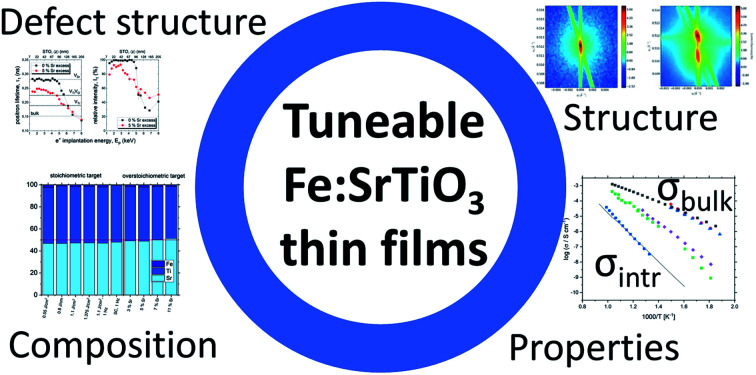

## 1. Introduction

SrTiO_3_ (STO) single crystals and thin films are currently a cornerstone for the study of multiple phenomena such as resistive switching in memristive devices,^1–4^ photoconductivity,^5^ increased oxygen incorporation under UV light,^6,7^ photovoltages in bulk SrTiO_3_,^8,9^ and interfacial conductivity, especially with LaAlO_3_.^10,11^ However, SrTiO_3_ thin films are not fully understood yet and show great variability in properties depending on the fabrication conditions. For instance, thin films prepared by pulsed laser deposition (PLD) showed that the film structure and composition depends on the deposition parameters, particularly on the laser fluence^12–15^ and on the oxygen partial pressure.^14^ The combination of different effects such as scattering of elements in the plasma plume and incongruent ablation may cause substantial cation non-stoichiometries leading to either Ti-rich thin films at higher fluences (due to preferential ablation of Ti species) or, opposite, Sr-rich thin films at low fluences (due to enhanced scattering of Ti).^15^ Stoichiometric thin films are reported when using a laser fluence of 1.1 J cm^−2^.^15^ Overall, the existing literature clearly indicates that the preparation of truly stoichiometric films by pulsed laser deposition is far from trivial.

Despite the numerous studies performed so far, there is still only a limited understanding of the structure–composition–property relations in SrTiO_3_ thin films. For example, it remains unclear how the crystal structure, the defect structure and the electrical properties of SrTiO_3_ thin films are correlated and how much those properties differ from the values found for single crystals. Particularly, charge transport properties might be very sensitive to changes in the point defect chemistry (*e.g.* due to cation non-stoichiometry) and thus pulsed laser deposited thin films may behave electrically very different compared to bulk samples. For example, a correlation between conductivity and lattice constant was found for Nb:SrTiO_3_ thin films.^16,17^ Other studies on nominally 0.4 mol% Fe doped SrTiO_3_ thin films (100 to 413 nm thickness) showed conductivities with values being orders of magnitude lower than those of the corresponding targets.^18,19^ Interestingly, these conductivities were very close to those expected for hypothetical ultrapure electronically intrinsic SrTiO_3_. Similarly, conductivities of Nb-donor doped SrTiO_3_ thin films have been varied by orders of magnitude simply by modifying the laser fluence in PLD deposited layers, which was correlated with different lattice expansion and, thus, cation non-stoichiometries.^20^ Also the properties of conducting SrTiO_3_/LaAlO_3_ heterointerfaces have been proved to be strongly affected by small variations of the LaAlO_3_ composition.^21,22^

Owing to this sensitivity of properties to the exact composition and/or structure of the thin films, it is mandatory to have sensitive tools for quantitatively evaluating the quality of PLD films and to control the deposition parameters for a proper tuning of the layers. A common tool for evaluating the film quality is the analysis of out-of-plane lattice constants in high resolution X-ray diffraction (HR-XRD) measurements.^14,23–25^ In these measurements, additional reflections (indicating different out-of-plane lattice parameters of epitaxially grown films) can be interpreted in terms of the presence of cation vacancies,^14,26,27^ revealing the existence of severe cation non-stoichiometries, *i.e.* Sr or Ti vacancies. Regarding the control of the layers, a common approach for the optimization of the film composition or film structure is the modification of deposition parameters (laser fluence, oxygen partial pressure, *etc.*) such that deviations of lattice constants vanish.

In this work, we study Fe-doped SrTiO_3_ films deposited using different parameters and target stoichiometries to elucidate their effect on the resulting layers (cation stoichiometry, structure and conductivity). The cation non-stoichiometry in 2% Fe doped SrTiO_3_ thin films is studied using HR-XRD, inductively coupled plasma optical emission spectroscopy (ICP-OES), positron annihilation lifetime spectroscopy (PALS) and by measuring across plane conductivities by means of electrochemical impedance spectroscopy (EIS). Film properties are modified by varying the PLD deposition parameters (laser fluences ranging from 0.55 J cm^−2^ to 1.375 J cm^−2^) and by using non-stoichiometric targets (ranging from 3% to 11% Sr excess). The latter approach turns out to be highly efficient when aiming at stoichiometric Fe:SrTiO_3_ films with conductivities close to those of macroscopic bulk Fe:SrTiO_3_. The sensitivity of the different tools for quantitative evaluation of the cation non-stoichiometry is compared.

## 2. Experimental

### 2.1 Thin film preparation

Fe-doped SrTiO_3_ (Fe:STO) thin films were prepared on different substrates by pulsed laser deposition (PLD). The laser source was a 248 nm KrF excimer laser (COMPex Pro 201F, Coherent, The Netherlands) with a pulse duration of 25 ns. The system was operated at two different pulse frequencies, 5 and 1 Hz, respectively. Laser fluences ranging from 0.55 J cm^−2^ to 1.375 J cm^−2^ were achieved by using nominal laser energies between 200 and 500 mJ. In the following, unless specified differently, a combination of 5 Hz and 1.1 J cm^−2^ is used (see ref. 15). In all cases, substrate temperature and oxygen partial pressure were 650 °C and 0.15 mbar, respectively, with a substrate to target distance of 55 mm. Not surprisingly the film thickness changes with changing laser fluence, laser frequency or target composition. In this study, deposition times were adapted such that all resulting films were of similar thickness for the impedance measurements, *i.e.* in the thickness range of about 150 to 350 nm for electrochemical measurements. Film thicknesses were measured by means of profilometry. Polycrystalline Fe doped SrTiO_3_ with 2% Fe per SrTiO_3_ unit cell was used as target material for most films, see below. Deposition parameters are summarized in Table 1.

**Table 1:** Pulsed laser deposition (PLD) parameters used for the preparation of the Fe-doped SrTiO_3_ thin films. Temperature and substrate material are the same for all samples, only frequency and – most importantly – laser fluence were varied. As a substrate, Nb:SrTiO_3_ (0.5 wt% Nb) was used for electrochemical characterization and positron lifetime annihilation spectroscopy, MgO and STO were used for XRD and RSM, respectively, and MgO was used as a substrate for chemical analysis

Parameter set	I		II	III	IV	V	VI	VII	VIII	IX	X
Fluence [J cm^−2^]	0.55	0.74	0.825	1.1	1.375	1.1	1.1	1.1	1.1	1.1	1.1
Frequency [Hz]	5	5	5	5	5	1	1	5	5	5	5
Pressure [mbar O_2_]	0.15	0.15	0.15	0.15	0.15	0.15	0.15	0.15	0.15	0.15	0.15
Temperature [°C]	650	650	650	650	650	650	650	650	650	650	650
Target	Stoichiometric polycrystal (PC)					Single crystal (SC)	PC	3% Sr excess PC	5% Sr excess PC	7% Sr excess PC	11% Sr excess PC

### 2.2 Polycrystalline targets

The polycrystalline targets were prepared by a mixed oxide route, starting with SrCO_3_, TiO_2_ and Fe_2_O_3_ (Sigma Aldrich, Germany). Stoichiometric as well as nonstoichiometric (3, 5, 7, and 11% Sr excess compared to the sum of Fe and Ti) amounts of the solids were accurately weighed, thoroughly mixed in an agate mortar and calcined at 1000 °C for 2 h under ambient conditions. For a stoichiometric cation composition (*i.e.* for a Sr amount corresponding to the amount of Ti + Fe), this preparation step already led to phase pure SrTiO_3_ powders. Afterwards the powders were crushed, cold isostatic pressed and sintered at 1200 °C for 5 h in air, resulting in targets used for PLD. The nonstoichiometric powders were calcined under the same conditions, but showed several different phases after the 1000 °C calcination. Nonetheless, the powders were crushed, pressed and sintered at 1200 °C for 5 h (X-ray characterization still showed different additional phases) and at 1400 °C for 4.5 h, yielding a target material that consisted of SrTiO_3_ with some Sr_3_Ti_2_O_7_ as an additional phase, see Results. As no futile compounds are expected to form, no change in composition is expected.

### 2.3 Substrate material

Fe:SrTiO_3_ thin films for conductivity measurements were grown on 5 × 5 × 0.5 mm^3^ Nb-doped SrTiO_3_ (Nb:STO) single crystals (0.5 wt% Nb content, CrysTec GmbH, Germany). Owing to the high conductivity of Nb:SrTiO_3_, this substrate acts as an electrode and allows across plane conductivity measurements of the thin films. Moreover, slightly doped Nb:SrTiO_3_ has the advantage of a very similar lattice parameter compared to undoped or slightly Fe-doped SrTiO_3_,^2,20,28^ which favours an epitaxial growth of the layer. Films grown on Nb:SrTiO_3_ were also used for the reciprocal space mapping (RSM) measurements. For positron annihilation lifetime spectroscopy, 10 × 10 × 0.5 mm^3^ Nb:SrTiO_3_ substrates were used in order to get information on the same substrate-thin film combination used in electrical measurement without any possible influence of a change in substrate. Fe:SrTiO_3_ thin films grown on 10 × 10 × 0.5 mm^3^ MgO (CrysTec GmbH, Germany), on the other hand, were used for additional gracing incident X-ray measurements and 5 × 5 × 0.5 mm^3^ MgO (CrysTec GmbH, Germany) substrates were used for the chemical analysis by ICP-OES. The MgO substrate leads to polycrystalline thin films, allowing a clear identification of the SrTiO_3_ phase in XRD. Moreover, the chemical analysis of the films can be performed in a standard manner, *i.e.* by dissolving the layer without quantification problems due to some substrate dissolution. For comparison, also 5 × 5 × 0.5 mm^3^ yttria stabilized zirconia (YSZ, CrysTec GmbH, Germany) were used as a reference substrate for chemical analysis. Please note that in all cases the substrate size equals the deposited area. During deposition, custom-made sample holders were used to prevent deposition on the sides and backside of the samples. The point of cross-comparison of data obtained for different substrates is addressed in ESI 8.†

### 2.4 Chemical characterization of the SrTiO_3_ films

The information on the elemental composition became accessible by dissolving the films and subsequent analysis by means of solution based ICP-OES (iCAP 6500, Thermo Fisher Scientific, USA). The SrTiO_3_ films on MgO were dissolved in 3% v/v HNO_3_ and 0.3% v/v HF for 30 min at 25 °C. Quantification was done *via* external calibration with liquid standard solutions. For a more detailed description, see ESI 1.†

### 2.5 Structural characterization

Different types of X-ray measurements were used to get information on the phase purity of the target (measurement in Bragg–Brentano geometry, BB), phase purity of the thin films (measurement in grazing incidence geometry, GID) and on the lattice mismatch of SrTiO_3_ layers compared to the substrate (reciprocal space mapping, RSM). For the phase identification of SrTiO_3_ targets, a PANalytical XPert Pro (MPD) powder diffractometer was used. A 2*θ* angle range of 15 to 90° was scanned, allowing a clear identification of SrTiO_3_. GID thin film measurements and reciprocal space mapping (RSM) were done on an Empyrean multipurpose diffractometer (PANalytical, Germany). The diffractometer was equipped with a Cu-anode operating at 45 kV and 40 mA providing a wavelength of *λ* = 1.5406 Å (Cu K Alpha 1) and *λ* = 1.5444 Å (Cu K Alpha 2). A hybrid monochromator (2xGe(220)) with a ½ divergence slit and a 2 mm mask was located within the incident beam path. On the detector side, a PIXcel 3D detector in scanning mode with an anti-scatter slit of 7.5 mm and a 0.04 rad soller slit was used. For the gracing incidence measurements, again a range between 15 and 90° was mapped. Reciprocal space maps were conducted for the (002) reflex (2*θ* = 46.4752°) and the (113) reflex (2*θ* = 81.7327°). The scan range around these two reflexes was 1° 2*θ* with a step size of *ω* of 0.0015° and a continuous scan of 2*θ*, resulting in a measuring time of around 12 h.

### 2.6 Positron annihilation lifetime spectroscopy

Variable energy positron annihilation lifetime spectroscopy (VEPALS) measurements were conducted on Fe:SrTiO_3_ samples at the Mono-Energetic Positron Source (MePS) beamline at HZDR, Germany.^29^ A digital lifetime CrBr_3_ scintillator detector [51 mm diameter (2′′) and 25.4 mm length (1′′)] coupled to a Hamamatsu R13089-100 PMT was utilized. The detector was a μ-metal shielded and housed inside a solid Au casing. For the data acquisition a homemade software was employed, executed from a multi-channel digitizer (SPDevices ADQ14DC-2X) with 14 bit vertical resolution and 2GS/s horizontal resolution. The time resolution of about 0.210 ns was achieved. The resolution function required for spectra analysis uses two Gaussian functions with distinct shifts and intensities, which depend on the positron implantation energy, *E*_p_. All spectra contained at least 1 × 10^7^ counts. Typical lifetime spectrum *N*(*t*) is described by *N*(*t*) = Σ(1/*τ*_*i*_)*I*_*i*_ exp(−*t*/*τ*_*i*_), where *τ*_*i*_ and *I*_*i*_ are the positron lifetime and intensity of the *i*-th component, respectively (Σ*I*_*i*_ = 1). All the spectra were deconvoluted using the non-linearly least-squared based package PALSfit fitting software^30^ into two discrete lifetime components, which directly evidence localized annihilation at 2 different defect types (sizes; *τ*_1_ and *τ*_2_). The corresponding relative intensities reflect to a large extend the concentration of each defect type (size) as long as the size of compared defects is in the similar range. In general, positron lifetime is directly proportional to defects size, *i.e.*, the larger is the open volume, the lower is the probability and longer it takes for positrons to be annihilated with electrons.^31,32^ The positron lifetime and its intensity have been probed as a function of positron implantation energy *E*_p_, which is proportional to the positron implantation depth.

### 2.7 Electrical characterization

**Fig. 1:**
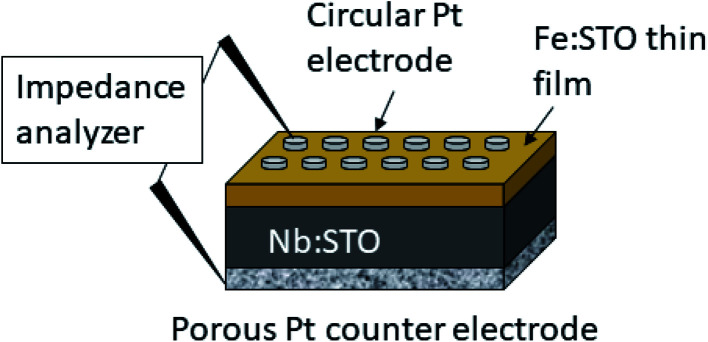
Sketch of the sample consisting on the Nb:SrTiO_3_ (Nb:STO) substrate, the Fe:SrTiO_3_ (Fe:STO) thin film and a porous Pt counter electrode as well as circular Pt microelectrodes on top.

The electrical characterization was performed by means of electrochemical impedance spectroscopy (EIS) on Fe:SrTiO_3_ films deposited on conducting Nb:SrTiO_3_. A sketch of the set-up is given in Fig. 1. Pt top layers were sputtered using a high voltage magnetron coating device (BAL-TEC MED 020, Germany) and microelectrodes with diameters in the range of 100–300 μm were prepared by lift-off photolithography. Pt paste (Tanaka Precious Metals, Japan) was brushed on the bottom side of the sample as a counter electrode. The Pt paste was pre-dried and then sintered at 850 °C for 2 h, resulting in a porous Pt electrode (see Fig. 1). Pt/Ir needles were used to contact the microelectrodes for impedance measurements.

Electrical measurements were then performed in a homogeneously heated furnace^33^ within a temperature range of 250 °C to 720 °C (though meaningful conductivities could be deduced for some films only in a limited temperature range)*.* Impedance spectra were obtained for frequencies from 0.9 MHz to 1 Hz with a resolution of 10 point per frequency decade, measured by an Alpha-A High Resolution Analyzer (Novocontrol, Germany). To ensure the probing in a linear regime, an AC rms amplitude of 20 mV was applied.

After electrical characterization, the films were checked for cracks or changes in the surface structure using optical microscopy. Furthermore, such Fe:SrTiO_3_ thin films were investigated using atomic force microscopy (AFM) and transmission electron microscopy (TEM) in previous studies.^18^

## 3. Results and discussion

### 3.1 Pseudo-intrinsic conductivity of films from stoichiometric targets

**Fig. 2:**
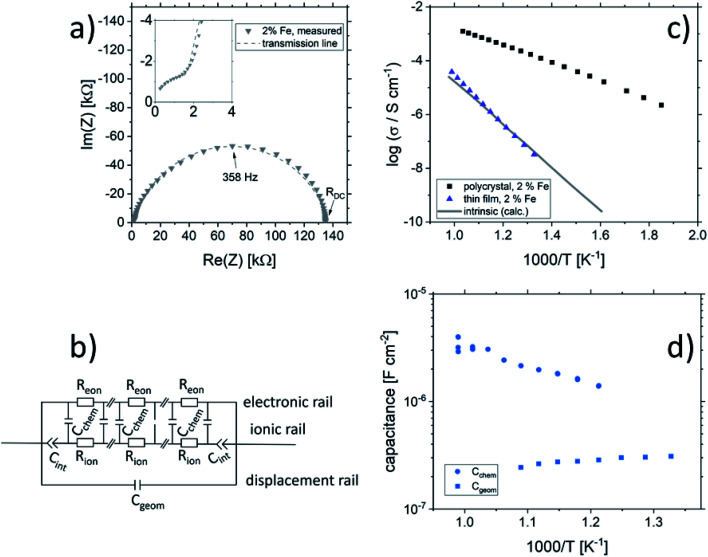
(a) Impedance spectrum of an Fe:SrTiO_3_ thin film deposited at 1.1 J cm^−2^ from a stoichiometric polycrystal with 2% Fe measured at 551 °C. Fitting was done with the transmission line model of a mixed conductor (b), consisting of an electronic rail, a blocked ionic rail and the displacement rail. The conductivity calculated from the DC resistance (=electronic resistance *R*_eon_) is plotted in an Arrhenius diagram in (c) and compared with the bulk conductivity of a polycrystalline pellet (2% Fe) as well as the calculated electronically intrinsic (*c*_e_ = *c*_h_) conductivity.^40^ Temperature dependent geometrical and chemical capacitance values for the 2% Fe doped film (370 nm thick) are shown in (d).

Fig. 2 shows a typical spectrum for SrTiO_3_ thin films prepared from 2% Fe doped stoichiometric targets. In addition to the somewhat distorted main arc, a well-separated much smaller arc appears at high frequencies. The permittivities deduced from the capacitance of the high frequency arc are very close to the expected SrTiO_3_ film bulk capacitances while the capacitance of the main arc is substantially larger (see also below). Typically, two separated arcs may either result from two serial processes, *e.g.* bulk transport and space charges at interfaces^34^ or from two parallel processes such as electron and ion conduction in a mixed conducting material, provided one of the two processes (paths) is blocked at the electrodes.^35^ SrTiO_3_ is known to be a mixed conductor^36^ and our set-up leads to a blocking of ions at one or both electrodes. Thus, use of a parallel path model is realistic here. More specifically, we employed the transmission line impedance model suggested in literature for such materials.^37–39^ This model consists of an electronic rail (*R*_eon_) and an ionic rail (*R*_ion_), which are connected *via* the chemical capacitance (*C*_chem_), and a displacement rail (*C*_geom_). The ionic rail is blocked by an interfacial capacitance (*C*_int_), as sketched in Fig. 2b. (In order to avoid over-parameterization, the same *C*_int_ is used for both electrodes.) This model excellently fits the measurement data of our 2% Fe-doped SrTiO_3_ films (see Fig. 2a). The number of fitting parameters is 6, namely *R*_ion_, *R*_eion_, CPE_int_ (*i.e. T*_int_, *P*_int_), *C*_chem_, and *C*_geom_. As a comparison, a parametrization using a serial fit with two R-CPE elements also results in 6 fitting parameters (*R*_1_, *T*_1_, *P*_1_, *R*_2_, *T*_2_, P_2_). For a discussion of the validity of the fit as well as a discussion on the error of the measurements as well as the fit used, see ESI 9–11.† Furthermore, three arguments considering the DC resistance as well as the resulting capacitances shown in Fig. 2d support the validity of the model and the corresponding data interpretation. Those are discussed in the ESI 2.†

An Arrhenius-type diagram of the resulting DC conductivity (*i.e.* electronic conductivity) of the thin films is shown in Fig. 2c. For comparison purposes, the electronic bulk conductivity of the corresponding 2% target material is also shown in Fig. 2c (the polycrystalline target leads to a bulk and a grain boundary arc in the complex impedance plane and only the bulk arc is considered here). As clearly visible in Fig. 2c, values of the 2% Fe-doped SrTiO_3_ thin film are three to five orders of magnitude lower than the expected ones (depending on temperature). Moreover, in accordance with literature data on 0.4% Fe doped films,^18^ the electronic conductivity of the 2% Fe films and its temperature dependence fit very well to that of intrinsic SrTiO_3_, calculated for the materials parameters from literature,^40^*i.e.* a band gap E_g_ of 3.3 eV − 6.0 × 10^−4^ eV × *T*, a hole mobility of 8.9 × 10^5^ (*T*/K)^−2.36^ cm^2^ V^−1^ s^−1^, an electron mobility of 4.5 × 10^5^ (*T*/K)^−2.2^ cm^2^ V^−1^ s^−1^ and *K*_0_ = 7.67 × 10^42^ cm^−6^. Here, “intrinsic” means identical hole and electron concentrations (*c*_e_, *c*_h_) as for a pure semiconductor without any further defect, *i.e. c*_e_ = *c*_h_ = 
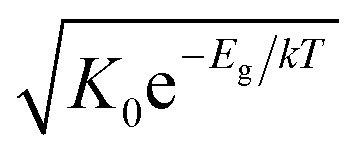
 (*k* = Boltzmann constant, *T* = temperature). Please note that electron and hole concentrations at 400 °C are thus in the range of 3 × 10^10^ cm^−3^ and in a usual situation this requires purity levels of semiconductors in the 0.01 ppb range. An activation energy of 1.75 ± 0.01 eV is found, fitting reasonably well to half of the band-gap energy, which is expected for an intrinsic material. An exact mechanistic explanation of this pseudo-intrinsic behaviour of Fe:SrTiO_3_ thin films, seemingly independent of the dopant concentration in the range between 0.4 to 2% Fe doping, is beyond the scope of this paper, but the following XRD and chemical analysis gives important empirical information on possible reasons.

**Fig. 3:**
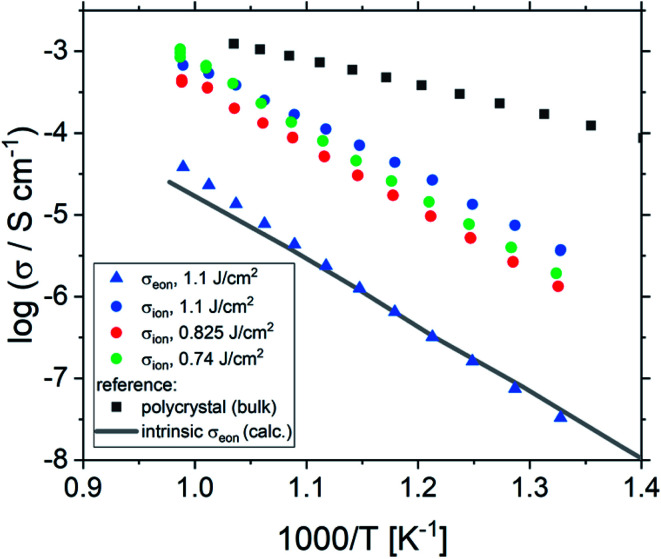
Electronic and ionic conductivities of SrTiO_3_ thin films deposited at 1.1 J cm^−2^ from 2% Fe doped stoichiometric polycrystals. Both are deduced from the transmission line fits. For comparison, ionic conductivities are also shown for other laser fluences. The intrinsic conductivity *σ*_eon_ was calculated using ref. 40.

Ionic conductivities of the same films are plotted in Fig. 3 showing one to two orders of magnitude larger values than the pseudo-intrinsic electronic conductivity. Here, an activation energy of 1.33 ± 0.01 eV is found. For a detailed discussion of activation energy for ionic conductivity, see below. Fig. 3 further displays variations of the ionic conductivities for films prepared with other fluences, see below. Different ionic defects are most probably also the reason behind differences in spectra shapes found between our 2% doped films and 0.4% Fe doped films.^18,19^ The yet not interpreted distortion of the spectra for 0.4% Fe thin films in ref. 18 and 19 thus probably comes from the mixed conduction. This is detailed in ESI 3.†

### 3.2 Structural, chemical and defect analysis of pseudo-intrinsic SrTiO_3_ films

**Fig. 4:**
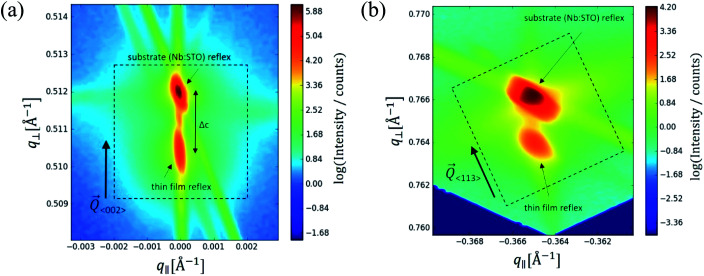
Reciprocal space mapping measurements for Fe:SrTiO_3_ standard thin films deposited from 2% Fe doped stoichiometric targets on Nb:SrTiO_3_ substrate material (PLD parameters [laser fluence, frequency, temperature, pressure, time]: 1.1 J cm^−2^, 5 Hz, 650 °C, 0.15 mbar and 20′). The out-of-plane measurement in close proximity of the (002) reflex is shown in (a). Arrows point at the substrate reflex as well as the thin film reflex. The differences indicating a lattice expansion Δ*c* are shown. The same sample was also measured around the (113) reflex (in-plane measurement). The resulting reciprocal space map is shown in (b). For clarification, arrows and dotted boxes indicate the direction of the diffraction vectors *Q*_〈002〉_ and *Q*_〈113〉_, respectively.

In the following, results from the structural, chemical and defect analysis of these pseudo-intrinsic SrTiO_3_ films are shown. First, we consider the XRD data of the films. Fig. 4 displays results from reciprocal space mapping (RSM) measurements on a thin film deposited with a repetition rate of 5 Hz and a fluence of 1.1 J cm^−2^. This high-resolution X-ray diffraction method can resolve deviations in the microstructure between the thin film and the substrate material.^41^ The diffraction vector *Q*_〈002〉_ normal to the surface is indicated. The detector streak (DS) as well as the monochromator streak (MS) can be attributed to the measurement set-up. The streak normal to the *q*_∥_ axis is the so-called crystal truncation rod (CTR), which is a diffraction phenomenon originating from the fact that the sample has finite dimensions.

The measurement around the symmetric (002) reflex (Fig. 4a) reveals a small deviation of the layer reflex in comparison to the substrate reflex. The substrate can be identified by the reflex position, which gives a lattice constant of 3.906 Å, in very good agreement with literature data for undoped SrTiO_3_.^28,42,43^ Moreover, the substrate reflex shows the highest count intensity. The thin film reflex (smaller count intensity) is slightly below the substrate reflex, indicating an expansion Δ*c* of the out-of-plane lattice constant *c*.

A relocation of the measuring spot on the asymmetric (113) reflex gives the reciprocal space map shown in Fig. 4b. Again, an arrow indicates the diffraction vector *Q*_〈113〉_. The measurement was performed using a gracing incident angle giving negative values for *q*_∥_. The lattice expansion of the thin film normal to the layer|substrate interface is confirmed by this measurement. In case of the asymmetric measurement, additional information on the in-plane lattice constant can be extracted. For the sample in Fig. 4b, the lattice parameter of the thin film parallel to the surface is perfectly matching the substrate, *i.e.* the layer reflex is located at the position *r* = 0. This indicates that the layer undergoes a pseudomorphic growth on top of the single crystalline SrTiO_3_ substrate.

Hence, we find a film growth with in-plane lattice constants as for the substrate, but significantly different out of plane lattice constants of substrate and film. This phenomenon is well-known in literature^12,13,44^ and is commonly attributed to the existence of cation vacancies, *i.e.* to films with a Sr/(Ti + Fe) ratio differing from one.^27,45–47^ For example, Wicklein *et al.* report that a broad range of different target materials lead to thin films showing either a reduced Sr-content, meaning that eihter Sr vacancies or Ti vacancies are present in the thin films.^15^ Furthermore, adding Fe to the target material can cause a very different ablation. The ablation behaviour changes due to an increased thermal conductance of the target,^48,49^ which then causes an increased heat penetration depth and a different dissipation of introduced laser energy.^49^ This leads to less material being ablated when Fe is present in the target material. Further mechanistic reasons for this non-stoichiometric deposition are discussed in ref. 48–50. In accordance with all the known literature we thus conclude that also our films exhibit a cation non-stoichiometry.

The estimated cation non-stoichiometry of the film was quantified by chemical analysis of the films. The quantitative data of the ICP-OES analysis of a standard film on MgO is shown in Table 2 and Fig. 6. The film shows a drastically reduced Sr content. Assuming that Fe occupies the B-site, we find an A/B site ratio in the film of 0.90 and thus a Sr deficiency in the range of 10%. Here, the error is well below 1% for main components (Sr, Ti) and in the range of 5% for the Fe dopant, *e.g.* 0.02 ± 0.001.

**Table 2:** Composition of Fe:SrTiO_3_ thin films prepared from stoichiometric targets by different deposition parameters and measured by ICP-OES. A pronounced Sr deficiency was found for all samples prepared from stoichiometric targets with 2% Fe and from the single crystal (SC) with 0.16% Fe

	Sr	Ti	Fe	Sr/(Ti + Fe)
PC, 2% Fe, 0.55 J cm^−2^, 5 Hz	0.94	1.01	0.051	0.88
PC, 2% Fe, 0.825 J cm^−2^, 5 Hz	0.94	1.04	0.023	0.88
PC, 2% Fe, 1.1 J cm^−2^, 5 Hz	0.94	1.02	0.035	0.90
PC, 2% Fe, 1.375 J cm^−2^, 5 Hz	0.95	1.03	0.027	0.90
PC, 2% Fe, 1.1 J cm^−2^, 1 Hz	0.94	1.02	0.014	0.89
SC, 1.1 J cm^−2^, 1 Hz	0.96	1.03	0.008	0.92

**Fig. 5:**
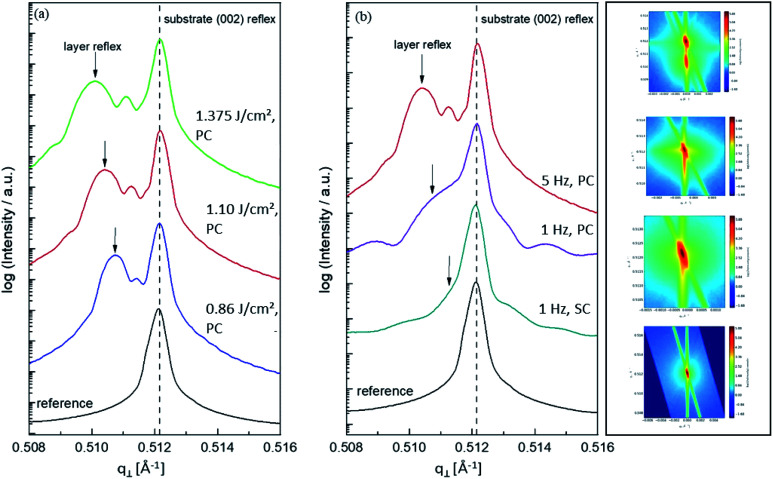
*ω–*2*θ* scans for various thin film samples. In (a), a comparison of SrTiO_3_ films (2% Fe doped stoichiometric targets) prepared with different laser fluences is shown (all other deposition parameters are held constant). The HR-XRD measurements were conducted around the (002) reflex. A black line indicates that the substrate reflex is in the same position for every sample. As reference the pure Nb:SrTiO_3_ substrate was measured. Arrows indicate the substrate reflex and illustrate the shift of these reflexes for changing deposition parameters. In (b), the laser fluence was kept constant at 1.1 J cm^−2^. Varying laser frequency (1 and 5 Hz) as well as target material (polycrystalline, 2% Fe doped, stoichiometric and single crystalline targets) result in thin films with a distinctly decreased lattice expansion. (Shifting of the layer reflex towards the substrate reflex.) The four RSM images belong to the four curves in (b).

**Fig. 6:**
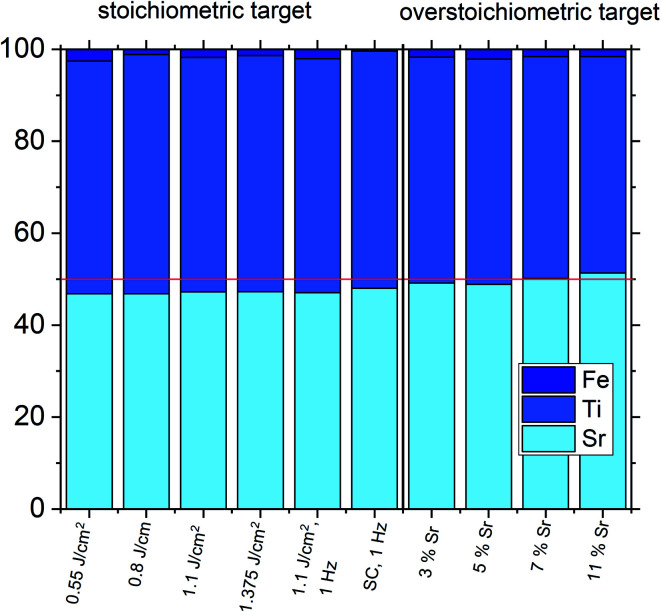
Composition of different SrTiO_3_ thin films on MgO measured by ICP-OES highlighting differences in the Sr content for different deposition parameters (targets without Sr excess: 2% Fe in PC, 0.16% Fe in SC) and for different Sr excess in the target. A stoichiometric thin film is only obtained by using a target with a 7% Sr overstoichiometry. At 11% Sr excess, a slight Sr excess is present in the thin film. In all other cases, a Sr deficiency can be seen. Fe is counted as B site ion in all cases.

**Fig. 7:**
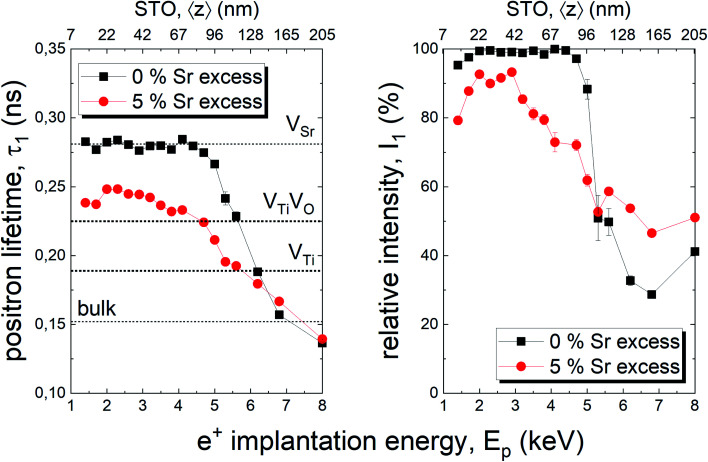
Positron annihilation lifetime spectroscopy measurements on Fe:SrTiO_3_ thin films deposited from 2% Fe doped targets with different cation stoichiometry on Nb:SrTiO_3_ substrates (5 Hz, 1.1 J cm^−2^). The thin film deposited from a stoichiometric target (labeled 0% Sr excess) clearly shows a positron lifetime which fits excellently to Sr vacancies. For larger implantation energies, transition to bulk values is seen. For films prepared from targets with 5% Sr excess, V_Ti_V_O_ clusters are found as the predominant defect type present in the respective thin film, highlighting a severe transition in defect chemistry induced by the introduction of Sr excess to the PLD targets.

In addition, positron annihilation lifetime spectroscopy (PALS) was performed and the implantation energy dependent positron lifetime *τ*_1_ is shown in Fig. 7. The positron lifetimes for lower energies probe the thin film and reveal Sr vacancies as predominant cation based point defect species for the thin film deposited from a stoichiometric target. Positron lifetime *τ*_1_ basically overlaps with the calculated literature value of 281 ps for Sr vacancies.^13^ At the same time, the relative intensity *I*_1_ is close to 100% indicating that Sr vacancies are the most abundant defect type, a typical scenario of large defect concentrations. For larger implantation energies, we see the transition to bulk properties. For films with 5% Sr excess, the film related positron lifetime decreases close to a literature value of *τ*_1_ = 225 ps indicating the emergence of a new smaller dominant defect type, arguably a complex of Ti vacancies and oxygen vacancies.^12,51^ (Please note that a Sr vacancy is a larger and stronger positron trap). Concomitantly a drop in *I*_1_ intensity reflects a larger chance for positrons to diffuse, as a consequence of a lower number of vacancy traps, and to annihilate with surface states.

Accordingly, the cation vacancies detected by RSM can be attributed to the A-site. In literature, Sr deficiencies in this range and similar out of plane deviations of lattice parameters were reported.^27,45–47^ Interestingly, also the Fe content detected in the films was consistently too high, indicating that the PLD process led to an enrichment in Fe, at least in the centre part of the plasma plume used to deposit the films. Measurements showed that indeed an inhomogeneous cation composition is expected in the PLD plasma used for SrTiO_3_ deposition and thus deviation of cation compositions compared to the target are not surprising.^15,44^ Similar non-stoichiometries introduced by the PLD process are reported in literature for other materials as well.^52–62^

We consider it as most plausible that such a severe A/B site non-stoichiometry in the SrTiO_3_ film is the origin of the pseudo-intrinsic film conductivity. We think that interplay of the Fe dopant (Fe acceptor states), a deep electron trap (possibly site defects,^63^*e.g.* Ti on the A-site^64–66^ acting as a donor) as well as a mid-gap state (Sr-vacancies^67^) causes a pinning of the Fermi level close to the centre of the band gap. Different gap states have also been reported for nominally undoped SrTiO_3_ thin films.^68^ However, a detailed mechanistic discussion of this phenomenon needs further investigation and is beyond the scope of this paper.

All together, the complementary tools used here (conductivity measurements, RSM, chemical analysis by ICP-OES, and PALS) directly or indirectly reveal the strongly non-ideal character of the grown SrTiO_3_ films (non-ideal in the sense of non-bulk SrTiO_3_ like). In the following, we discuss how this non-ideality can be reduced or even eliminated and how sensitive the tools are to monitor cation non-stoichiometry of the film.

### 3.3 SrTiO_3_ film tuning by controlling the deposition conditions (stoichiometric targets)

One common strategy for reducing the cation non-stoichiometry is the variation of the deposition parameters in PLD grown layers. It is reported that lowering the laser fluence reduces the Sr vacancy concentration.^14,15,23^ In this work, a range of laser fluences was employed, from 0.55 J cm^−2^ to 1.375 J cm^−2^ (Section 3.1 reports on 1.1 J cm^−2^). Moreover, different laser frequencies were employed (1 and 5 Hz) and also a Fe:SrTiO_3_ single crystal was used as the target (though with a lower Fe content of 0.16% Fe). In Fig. 5, rocking curves for thin films deposited using different laser fluences (0.86 J cm^−2^ to 1.375 J cm^−2^), repetition rates (1 Hz, 5 Hz) and target materials (polycrystal, single crystal) are shown and reveal that the higher laser fluence indeed enhanced the out of plane lattice mismatch while lower laser fluence lowered the mismatch. However, within the given fluence range and for 5 Hz deposition rate a clear indication of a lattice mismatch remained. Reducing the frequency lead to thinner films and thus less pronounced signals but the lattice mismatch itself remained and therefore also indication of the film non-stoichiometry. The thin film prepared from the 0.16% doped Fe:SrTiO_3_ single crystal showed least indication of a cation non-stoichiometry in RSM curves.

**Fig. 8:**
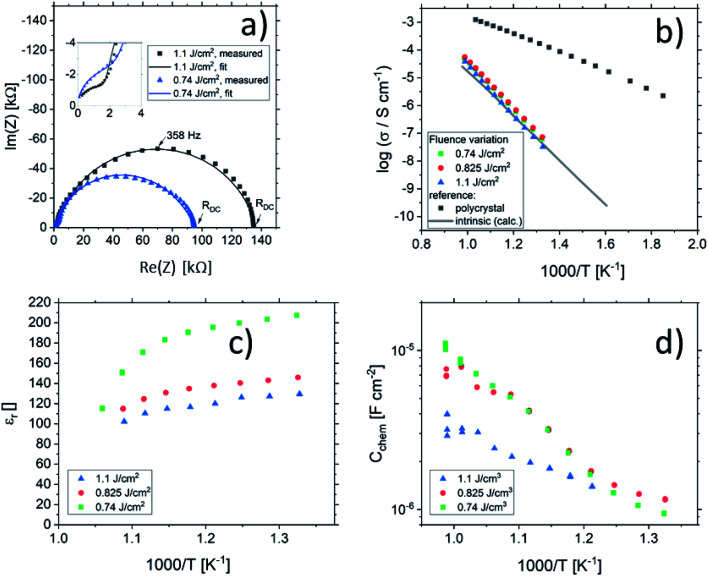
Impedance spectra of STO thin films deposited from 2% Fe doped stoichiometric targets using different laser fluences (1.1 J cm^−2^ and 0.74 J cm^−2^) at 551 °C and 553 °C, respectively, showing a strong distortion for both semicircles (a). The dc (=electronic) conductivities of thin films deposited at different laser fluences are plotted in an Arrhenius plot (b) and are compared to the bulk conductivity of a polycrystal and the electronic intrinsic conductivity calculated using ref. 40. Bulk permittivity values and chemical capacitances deduced from the transmission line model are shown in (c) and (d), respectively.

Data from the chemical analysis of the films are summarized in Table 2 and Fig. 6 and reveal that the pronounced Sr deficiency is not much affected by the different preparation conditions. Also the film prepared from the single crystal exhibits a slightly smaller but still severe A-site deficiency. In all samples, again more Fe is found than in the target and on average the Fe content is almost twice the nominal one, most likely due to plume-related processes, such as preferential scattering, or plume-background interactions.^14,15,58^ The impedance spectra were similar to those of the films in Section 3.1, but the distortion of the main arc became even more prominent in some cases (see Fig. 8a). The fitting procedure for these impedance spectra was carried out as described in Section 3.1, *i.e.* with a transmission line based model, and the electronic conductivity, ionic conductivity, relative permittivity and chemical capacitance were deduced. Again, the DC resistance corresponds to the electronic conductivity. The ionic conductivity (Fig. 3) as well as the chemical capacitance (Fig. 8d) show some fluence dependency. The moderate deviation in bulk permittivity (from *C*_geom_) are probably due to non-idealities of the fit model. However, the electronic conductivity does not change due to the different deposition parameters (Fig. 8b). Rather, again pseudo-intrinsic conductivity values were found for the samples investigated (0.74 and 0.825 J cm^−2^). Activation energies for the electronic/total conductivity of 1.71 ± 0.01 eV and 1.73 ± 0.01 eV for 0.825 J cm^−2^ and 0.74 J cm^−2^ are found, which are well in line with the activation energy found in the thin film deposited at 1.1 J cm^−2^. For ionic conductivity, activation energies of 1.57 ± 0.01 eV and 1.58 ± 0.01 eV are found, respectively. In literature, for ionic conductivity in undoped or slightly Fe- or Ni-doped SrTiO_3_ single crystals, an activation energy of 0.9 is reported,^40^ which is significantly lower than the activation energies found in this study. However, interactions between dopants and oxygen vacancies are known to increase, for example, the migration enthalpy from 0.62–0.67 eV up to 1 eV.^69^ In our case, the extremely high amount of Sr vacancies might affect oxygen transport through defect association with oxygen vacancies, thereby also affecting activation energies.

### 3.4 SrTiO_3_ film tuning using different target compositions

**Fig. 9:**
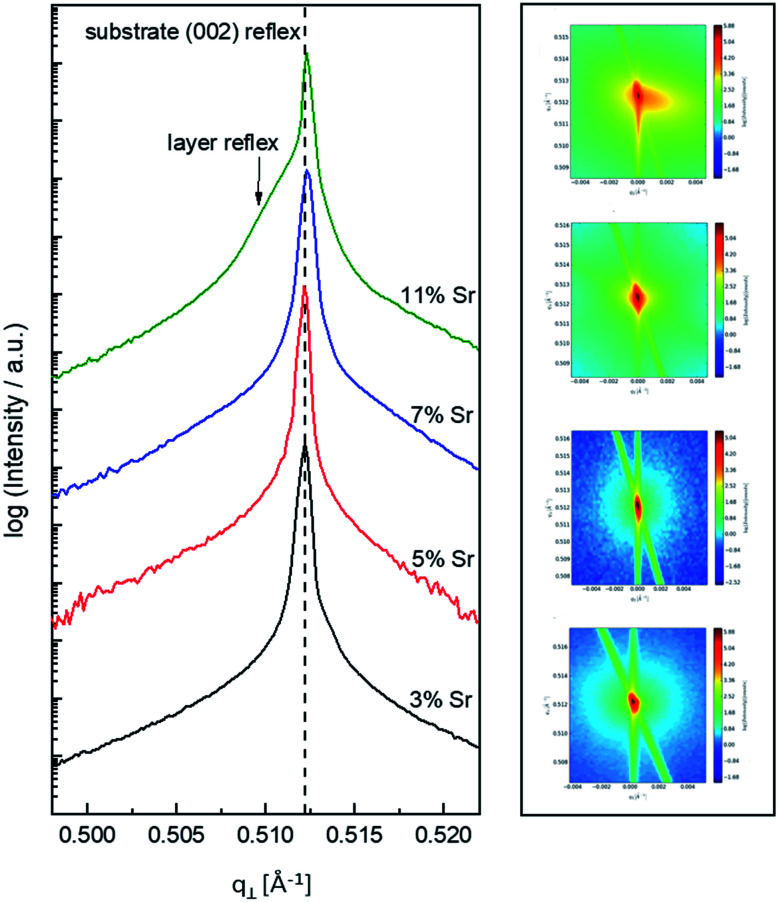
*ω*–2*θ* scans for various thin film samples deposited from 2% Fe-doped SrTiO_3_ polycrystalline targets with different Sr overstoichiometry. The HR-XRD measurements were conducted around the (002) reflex. The black dashed line indicates that the substrate reflex is in the same position for every sample. Laser fluence was kept constant at 1.1 J cm^−2^. At the right hand side, the corresponding reciprocal space maps are shown as well.

Based on the knowledge that Sr deficiencies (in the range of several percent) result in films deposited by PLD, we prepared different targets with a Sr surplus of 3 to 11% (details on the target preparation are given in the experimental part). XRD of these targets clearly revealed the Sr over-stoichiometry by reflexes from a second phase, namely Sr_3_Ti_2_O_7_, and its amount increased with Sr content, see ESI 4.† The films grown from these targets, however, did not show any indication of a secondary phase, neither in XRD measurements of polycrystalline films on MgO (see ESI 5†) nor in RSM measurements (see Fig. 9). Rather, their reflections could not be distinguished from the substrate reflections in rocking curves, at least for 3–7% Sr, see Fig. 9. Only 11% Sr surplus again caused a shoulder in the RSM curve which is most probably due to Ti vacancies in this case.

On the basis of XRD data, all three films with 3, 5 and 7% Sr surplus are compatible with stoichiometric compositions. This conclusion, however, was only partly confirmed by the chemical analysis. Table 3 and Fig. 6 show the results from ICP-OES measurements. Indeed, the A/B site ratio was much closer to unity for those three films compared to films prepared from the stoichiometric target. However, for 3 and 5% Sr excess the nominal A/B site ratio is still only 97 or 96%, respectively (with errors well below ±1%). The even slightly lower nominal value for 5% Sr excess compared to 3% Sr is within the error bar. Moreover, in these calculations, Fe is always counted as a B site ion, which is not necessarily the case if many A-site vacancies are present. The 7% Sr film, on the other hand, showed almost exact A/B site stoichiometry in ICP-OES measurements, though still more Fe is present than in the target. For 11% Sr excess, A/B of 1.06 is measured (see Table 3), in accordance with the shoulder in the rocking curve indicating too much Sr (see Fig. 9). Please note, that most of these films have Sr/Ti ratios which can hardly be distinguished by XPS or XRD studies with typical errors in the 5% range.^14,70,71^

**Table 3:** Composition of differently prepared Fe:SrTiO_3_ thin films, deposited from overstoichiometric targets and measured by ICP-OES. The Sr excess in the target material counterbalances the loss during the PLD process, leading to A : B-ratios closer to unity

	Sr	Ti	Fe	Sr/(Ti + Fe)
2% Fe, 0% Sr, 1.1 J cm^−2^	0.94	1.02	0.035	0.90
2% Fe, 3% Sr, 1.1 J cm^−2^	0.98	0.98	0.034	0.97
2% Fe, 5% Sr, 1.1 J cm^−2^	0.98	0.98	0.043	0.96
2% Fe, 7% Sr, 1.1 J cm^−2^	1.00	0.96	0.032	1.01
2% Fe, 11% Sr, 1.1 J cm^−2^	1.03	0.94	0.032	1.06

**Fig. 10:**
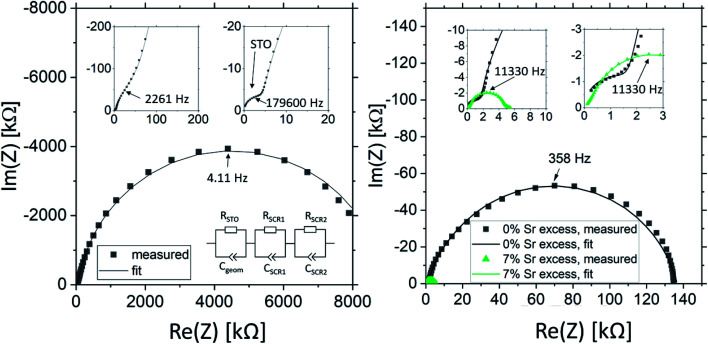
(a) Impedance spectrum of a thin film deposited from a 2% Fe doped target with 7% Sr overstoichiometry measured at 327 °C. The magnification reveals a medium frequency shoulder and an additional small high frequency semicircle. Only the high frequency semicircle (*R*_STO_) is attributed to the Fe:SrTiO_3_ bulk according to the geometrical capacitance. The medium frequency shoulder and the big low-frequency semicircle are attributed to space charge regions (*R*_SCR1_, *R*_SCR2_). (b) Impedance spectrum of the same thin film measured at 556 °C and the respective impedance spectrum of a thin film deposited from a stoichiometric target as a reference (measured at 551 °C), highlighting the dramatic change in absolute resistance.

The impedance spectra of these films are very different from those of the pseudo-intrinsic standard films. As an example, we show a spectrum for the 7% Sr excess case in Fig. 10. At 330 °C, it becomes obvious that the spectrum consists of three arcs with dramatically different sizes, a very small high frequency arc or shoulder, a medium sized medium frequency arc and a very large low frequency arc (see Fig. 10a). Hence, compared to the pseudo-intrinsic standard films, an additional feature appears. However, the total resistance of the large low frequency arc is still much smaller than the main arc measured for the standard films prepared from stoichiometric targets. This is illustrated by comparing spectra of the pseudo-intrinsic standard films with the films from 7% Sr excess targets measured at 555 °C (see Fig. 10b). At this temperature, only the low and the medium frequency arcs are still visible in the measured frequency range of the 7% excess film. The entire dc resistance of the 7% Sr excess film (sum of three arcs) is not much larger than the high frequency arc of the pseudo-intrinsic film. In general, the high frequency arc of the 7% Sr excess films is orders of magnitude smaller than that of the standard films. This strongly indicates that also a reinterpretation of the spectra is required for these Sr-excess films. The transmission-line based rail model does not fit the data anymore, and a physical meaning must be found for the appearance of an additional feature.

The most straight-forward fitting approach is to use a serial fit. A fit to three serial R-CPE elements works very well and the interpretation of the spectra is done based on the corresponding capacitances. (Please note that 8 to 9 free parameters were used in total, *i.e. R*_1_, *T*_1_, *P*_1_ (usually fixed to one), *R*_2_, *T*_2_, *P*_2_, *R*_3_, *T*_3_, and *P*_3_ with *P*-parameters between 0.9 and 1.) The capacitance of the high frequency arc (visible in Fig. 10a) fits well to the geometrical capacitance expected for the entire SrTiO_3_ film, *i.e.* it leads to a very reasonable permittivity in the range of 150.^72^ Hence, this arc is attributed to the total bulk conductivity of the corresponding SrTiO_3_ thin films. The other two capacitances are about 10 and 20 times larger than the high frequency capacitance and thus correspond to SrTiO_3_ layer regions in the 10 or 20 nm range assuming bulk permittivity. Those are most probably interfacial space charges at the two electrodes, see also ESI 6.† Those come into play since the electronic conductivity is no longer at its lowest possible value (intrinsic). Accordingly, the ionic conductivity plays no longer a role in this interpretation. Hence, the serial fit is not only a parametrization of the spectra, but is to our understanding physically meaningful and therefore justified. For a detailed discussion on the error of the measurement and the fit, see ESI 9.†

**Fig. 11:**
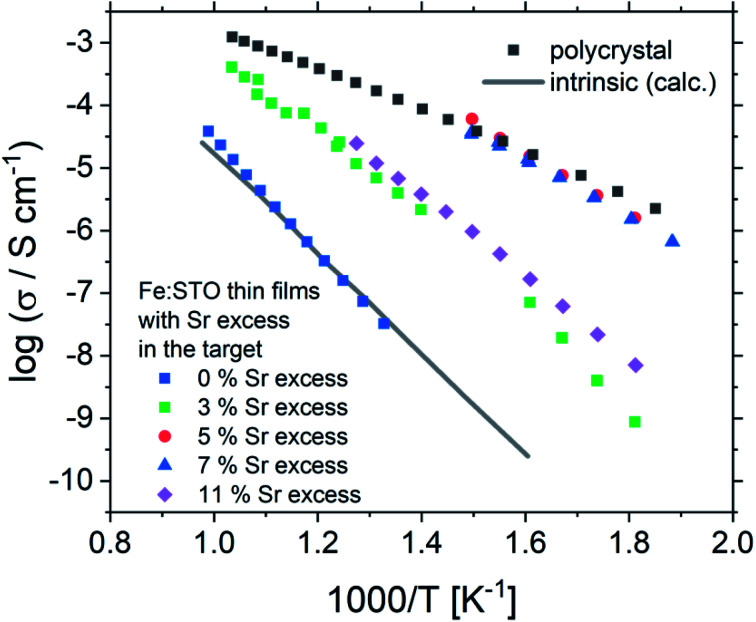
The total conductivities of Fe:SrTiO_3_ thin films deposited from targets with 2% Fe and different Sr overstoichiometry are plotted in an Arrhenius diagram and compared with the electronic bulk conductivity of a polycrystal as well as the electronic intrinsic conductivity calculated using ref. 40. Here, the increase in conductivity of the samples with 5% and 7% Sr overstoichiometry up to electronic bulk conductivity of a polycrystal can be seen.

Based on this interpretation, the high frequency arc is used to determine the bulk conductivity of these thin films. For the other Sr excess cases, the spectra have also a high frequency arc with bulk-like capacitance, though partly only one additional interfacial arc is visible. The same type of analysis is thus performed also for the other films. These bulk conductivities of the films prepared from over-stoichiometric targets are shown in Fig. 11. The film prepared from 3% Sr excess target exhibits bulk conductivities which are higher than the electronic ones of pseudo-intrinsic films (and similar to ionic conductivities of those), but those are still far off the expected electronic bulk conductivity of polycrystalline SrTiO_3_. Hence, the improved cation stoichiometry is sufficient to strongly reduce structural deformations (*c.f.* the rocking curves in Fig. 9), but defect chemical non-idealities are still so large that orders of magnitude lower electronic conductivities are found. This also shows that XRD curves have reduced sensitivity towards off-stoichiometries, *i.e.* they may indicate severe deviations from the desired cation stoichiometry while absence of rocking curve shoulders cannot be taken as an indication of excellent stoichiometry or defect chemical ideality.

The films prepared from 5% Sr and 7% Sr excess, however, show bulk conductivities very close to the expected bulk electronic conductivity of pellets. This agreement supports the interpretation that the total bulk conductivity in the thin film is now largely electronic, as in the bulk sample. Hence, stoichiometry deviations occurring during the deposition process seem to be largely counter-balanced by the Sr excess in the target. In the case of the sample prepared from the 7% Sr excess, the bulk-like behaviour of the thin film is also in agreement with the excellent stoichiometry revealed by ICP-OES. For the 5% Sr-excess the agreement between film and bulk pellet is even a bit surprising since the exact stoichiometry is still not hit: A site deficiency is present with a rather large Fe content (4.3% Fe). However, those deviations (Sr deficiency and Fe excess) might partly counterbalance each other for example by Fe on the A site. Transition metal ions on the A site are, for example reported also for Mn.^73^ In any case, the rather ideal conductivity also fits excellent to the PALS experiment, where for 5% Sr excess no Sr vacancies, but instead associates of titanium vacancies and oxygen vacancies (V_Ti_V_O_) are found, compared to the Sr vacancies for the thin film prepared from a stoichiometric target (see Fig. 7). The conductivity of the films with 11% Sr excess is again rather low, but still larger than that of pseudo-intrinsic SrTiO_3_ thin films. In terms of activation energies a trend towards lower activation energies for bulk-like thin films is observed and values of 1.38 ± 0.04 eV, 0.99 ± 0.01 eV, 0.91 ± 0.01 eV and 1.29 ± 0.01 eV are found for thin films deposited from targets with 3%, 5%, 7%, and 11% Sr excess. For the sample deposited from a 7% excess target, the value perfectly matches literature data for grain conductivity.^74^ From this we can conclude that our bulk-like thin films indeed behave like SrTiO_3_ bulk samples and that any deviation from ideal stoichiometry leads to a more or less pronounced increase in activation energy up to about half of the bandgap.

All in all, our study reveals the importance of the deposition process in SrTiO_3_ thin films and the need for a characterization beyond XRD to ensure a proper stoichiometry and, therefore, functionality since a rather small deviations from the main cation stoichiometry strongly affects the conductivity. Here, we propose an efficient way for preparing stoichiometric Fe:SrTiO_3_ films by using Sr-rich targets (*i.e.* non phase-pure targets). Since it is known that PLD layers may strongly vary between different systems, the optimal Sr-excess found here might need readjustment in other equipment. Finally, a simple methodology for properly deciding whether a stoichiometric film has been prepared or not seems to be only found in the measurement of the conductivity at elevated temperature, yielding drastically lower conductivities even for rather small deviations from the cation stoichiometry.

## 4. Conclusions

Fe:SrTiO_3_ thin films prepared from stoichiometric targets show severe Sr deficiencies which are quantified by ICP-OES, revealing about 10% nominal A-site deficiency, but also enhanced Fe content. Off-stoichiometries are also clearly visible in RSM measurements by additional reflections due to different out-of-plane lattice constants (compared to single crystalline SrTiO_3_). Moreover, PALS measurements show the presence of Sr vacancies. The conductivity of such layers is many orders of magnitude lower than the electronic conductivity of the polycrystalline target material and almost perfectly matches the expected electronic conductivity of ultra-high purity intrinsic SrTiO_3_ (with mid-gap Fermi level). Variation of PLD deposition conditions affects the out-of-plane lattice constant measured in XRD measurements. However, chemical composition and conductivity of these films are not substantially changed. The use of Sr-rich targets enhances the Sr content in the deposited films and, especially, laser ablation of 7% Sr-excess targets results in films with the correct A/B cation stoichiometry. Moreover, these stoichiometric films exhibit conductivities almost matching the bulk conductivity of polycrystalline Fe:SrTiO_3_. Activation energies range from half of the band-gap (∼1.6 eV) for pseudo-intrinsic samples to the literature value for bulk-like samples (∼0.9–1 eV). The measurement of the conductivity is thus the most sensitive tool for finding the conditions for which stoichiometric films can be obtained. Opposite, RSM and out-of-plane lattice parameter analysis (often employed as single characterization) only indicates non-idealities for rather pronounced deviations from cation stoichiometry.

## Conflicts of interest

There are no conflicts to declare.

## Supplementary Material

NA-003-D1NA00358E-s001
