# Supplementary material for: Cation non-stoichiometry in Fe:SrTiO3 thin films and its effect on the electrical conductivity
Source: Nanoscale Adv. 2021 Sep 10;3(21):6114–27. doi: 10.1039/d1na00358e (PMC8548878; doi:10.1039/d1na00358e)
Supplement: NA-003-D1NA00358E-s001 [file NA-003-D1NA00358E-s001.pdf]

Electronic Supplementary Material (ESI) for Nanoscale Advances.
This journal is © The Royal Society of Chemistry 2021

Supplementary Information:

Cation non-stoichiometry in Fe:$SrTiO_3$ thin films and its effect on the electrical conductivity

Maximilian Morgenbesser[1], Stefanie Taibl[1], Markus Kubicek[1], Alexander Schmid[1], Alexander Viernstein[1], Niklas Bodenmüller[1],Christopher Herzig[1], Federico Baiutti[2], Juan de Dios Sirvent[2], Maciej Oskar Liedke[3], Maik Butterling[3], Andreas Wagner[3], Werner Artner[4], Andreas Limbeck[1], Albert Tarancon[2,5], Jürgen Fleig[1]

1) Institute of Chemical Technologies and Analytics, TU Wien, Getreidemarkt 9-164/EC, 1060 Vienna, Austria

2) Catalonia Institute for Energy Research (IREC), Jardins de Les Dones de Negre 1, 08930 Sant Adria del Besos, Barcelona, Spain

3) Institute of Radiation Physics, Helmholtz-Zentrum Dresden-Rossendorf (HZDR), Bautzner Landstraße 400, 01328 Dresden, Germany

4) Fachbereich Röntgenzentrum, TU Wien, Getreidemarkt 9, 1060 Vienna, Austria

5) ICREA, 23 Passeig Lluis Companys, Barcelona 08010, Spain

SI 1 ICP-OES measurements

To determine the actual composition of the $SrTiO_3$ thin films, inductively coupled plasma-optical emission spectroscopy (ICP-OES) was used. Therefore, samples with about 100 nm film thickness were deposited on 5x5 mm^2 YSZ single crystals. These layers were dissolved in 5 mL of 3 % (v/v) nitric acid (65 mass%, EMSURE®) and 0.3 % (v/v) hydrofluoric acid (40 mass%, Suprapur®). The dissolving agent was prepared by mixing deionised water obtained by Barnstead™ Easypure ™ II (18.2 M cm^{-1}), the concentrated acids and 1000 mg kg^{-1} Eu single element standard (Certipure®, Merck, Germany). The final concentration of Eu was adjusted to 1 mg kg^{-1} in the diluted acid mixture and was used as an internal standard to correct for possible signal drifts. After 30 min of dissolving time, the obtained sample liquid was transferred into a new polypropylene sample tube to remove the remaining substrate and to stop a possible dissolution process of the substrate. This whole process was conducted under ambient conditions at room temperature and the derived sample solutions were measured without any further dilution.

For signal quantification, single element standards (for details see Table 2) were mixed with the diluted acid mixture (3 vol% HNO_3, 0.3 vol% HF) already containing the internal standard to performe an external calibration. Standard solutions with varying concentration levels from 0.2 to 12.6 mg kg^{-1} for the main components Sr and Ti and 0.002 to 0.126 mg kg^{-1} for the Fe dopant were prepared. With the obtained signal intensities, regression lines were derived to calculate the analyte concentration of the unknwon samples.

Samples and standards were analyzed with an iCAP 6500 ICP-OES spectrometer (ThermoFisher Scientific, Bremen, Germany) equipped with a MiraMist nebulizer and a cyclonic spray chamber (Glass Expansion, Port Melbourne, Australia). Sample-uptake was achieved with the peristaltic pump of the instrument (25 rpm, 0.64 mm ID pump tubing). Background-corrected emission

signals were recorded in the radial viewing mode and processed using Qtegra software (Thermo Scientific, USA). Six replicates with an integration time of 10 s each were measured for samples as well as standard solutions. The optimized ICP-OES parameters and the monitored emission lines are summarized in Table 1. For each element several intense but non interfered emission lines were measured.

Observed signal intensities were normalized using the signal response for the internal standard (Eu), and finally converted into concentration units by means of the external calibration. By using the molar masses of each element, the mole fractions of the cations present in the investigated samples were calculated. Obtained Eu signals were constant over each measurement session (less than 5% relative standard deviation for the whole measurement period, indicating the absence of temporal trends), and no significant difference in Eu-response between samples and calibration standards was observed.

Tab. SI 1: Optimized ICP-OES parameters used for measurements.

RF power	1200 W
exposure time	10 s
nebulizer gas flow	0.75 L min^{-1} argon
type of nebulizer	MiraMist®
sample flow rate	0.7 mL min^{-1}
pump tubing	Tygon®, 0.64 mm ID (color code: orange-yellow)
cooling gas flow	12 L min^{-1} argon
auxiliary gas flow	0.8 L min^{-1} argon
viewing height above load-coil	10 mm
Spectral range	Visible
Elements	Emission line [nm]
Sr	421.55*, 346.45, 216.60
Ti	334.45*, 232.45, 338.38
Fe	259.94, 238.20*, 240.49
Eu	381.97*, 412.97

Emission lines marked with * used for evaluation.

Tab. SI 2: Single element standards used for signal quantification.

**Element**	**Product information, lot number**	**Concentration levels**

			**for calibration [mg kg^{-1}]**
Sr	Certipure®, Merck, Germany	HC87301854	0.2, 0.4, 0.8, 1.6, 3.2, 6.4, 12.8
Ti		HC99678363	
Fe		HC86803426	0.002, 0.004, 0.008, 0.016, 0.032, 0.064, 0.128
Eu	Specpure®, Alfa Aesar, Germany	35753	1.0

SI 2 Three arguments supporting the use of a transmission line based fitting model of impedance spectra obtained for thin films deposited from stoichiometric targets

We used a transmission line based fitting model with parallel ionic and electronic paths for impedance spectra of thin films deposited from stoichiometric targets. Here, three arguments are given supporting the validity of this approach. The first argument refers to the electronic resistance R_{eon}. This resistance is essentially the dc resistance and the resulting conductivity σ_{eon} is plotted in Fig. 2.c in the main paper. The conductivity found for the thin film deposited from a target with 2 % Fe doping exhibits an activation energy of 1.57 eV and agrees very well with the intrinsic conductivity of a hypothetical ultrapure $SrTiO_3$ film. Such an excellent agreement cannot be accidental and can hardly be understood from a simple serial impedance model. The second argument refers to the geometrical capacitance C_{geom} in the fit (which depends mainly on the high frequency arc) – cf. Fig. 2.d in the main paper. The permittivity deduced from this value is rather close to that of $SrTiO_3$ (ca. 140 – 180 [1], depending on temperature), which supports the appropriateness of the model. The chemical capacitance resulting from the fit gives a third argument and is also shown in Fig. 2.d in the main paper. It comes mainly from the large arc and is about one order of magnitude larger than the geometrical capacitance. Its significant increase with temperature is typical for chemical capacitances, which originate from oxygen stoichiometry changes and thus defect chemical processes in the material upon the application of an alternating voltage. (An even stronger temperature dependence is found for other fluences, see main paper Fig. 8.d). Hence, we conclude that the model is meaningful and the dc resistance is the electronic resistance (due to ion blocking electrodes). The high frequency arc, on the other hand, corresponds to the total conductivity of ions and electrons.

SI 3 Comparison with data from literature on films from 0.4 % Fe doped targets

The pseudo-intrinsic behaviour of $SrTiO_3$ thin films was already reported in Ref. [2] for films prepared from 0.4 % Fe doped stoichiometric targets. Interestingly, the rather pronounced high frequency shoulder found in our study on 2 % Fe doped films was not present for the 0.4 % Fe doped films discussed in Ref. [2, 3]. In order to exclude the relevance of artefacts and to further understand the different shape of the spectra, we also prepared 0.4 % Fe doped films here (1.1 J/cm^2, 5 Hz, investigated with $La_{0.6}Sr_{0.4}CoO_{3-\delta}$ microelectrodes prepared as described in Ref. [4].) All results shown in the earlier studies were excellently reproduced for these 0.4 % Fe:$SrTiO_3$ films. Fig. SI 1.a shows a typical EIS spectrum for 0.4 % Fe which consists of one depressed and asymmetric arc with a high frequency intercept, very similar to the earlier study. The conductivity calculated from the low frequency intercept of the main arc (i.e. the dc resistance, R_{DC}) is shown in the Arrhenius plot of Fig. SI 1.b. The values match very well to those of Ref. [2], i.e. they are again pseudo-intrinsic.

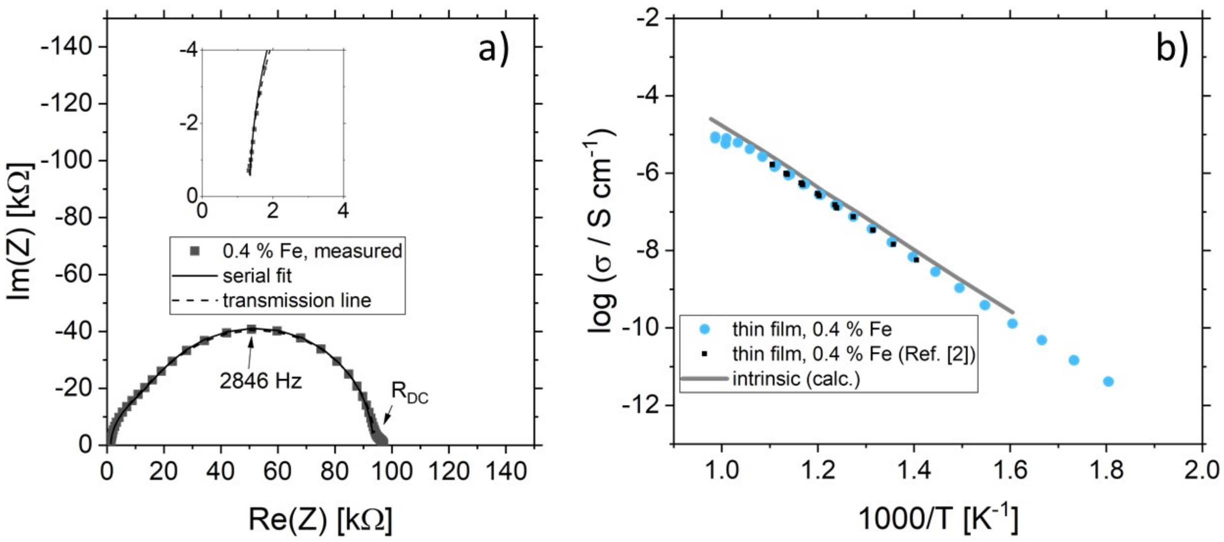

Fig. SI 1: (a) Impedance spectrum of a Fe:SrTiO$_3$ thin film deposited from a stoichiometric target with 0.4 % Fe doping. A distorted arc is visible. Fitting can be done with either using a serial fit or a transmission line base model, see below. The conductivity obtained from the DC resistance (highlighted using an arrow in (a)) is plotted in (b) for different temperatures and compared with the electronic intrinsic conductivity [5] as well as with literature data [2], showing great agreement to both.

The more or less distorted arc with a high frequency intercept found here and in Ref. [2] for 0.4 % Fe doping can be fitted by a serial circuit of a R followed by one or two R-CPE (CPE = constant phase element). The serial R is a contact resistance and is not further considered. For main arcs with little distortion, one R-CPE element is sufficient and the corresponding capacitance then corresponds very well to the expected bulk capacitance of the thin film. However, not surprisingly, also a fit is possible with the transmission line model suggested in the main paper and representing

the mixed conducting character of the $SrTiO_3$ films. Based on this model the small high frequency arc for 2 % Fe doped films indicates a total conductivity which is much higher than the pseudo-intrinsic electronic conductivity due to parallel ion conduction. The absence of the high frequency arc for 0.4 % Fe, on the other hand, thus suggests that either the ionic conductivity is very low and does not strongly enhance the total conductivity, or the corresponding chemical capacitance in the transition line model becomes too small to allow a separation of the arcs, or both.

We think that the appearance of some distortion in Fig. SI 1.a (0.4 % Fe) has essentially the same reason as the much smaller high frequency arc for 2 % Fe, namely an enhanced total conductivity due to ion conduction. However, for 0.4 % Fe doped films the enhancement is only minor since the corresponding ionic conductivity is still in the same range as the pseudo-intrinsic electronic conductivity or even lower. Thus, either only the distortion of the main arc or simply nothing at all indicates existence of some ion conduction in the film, in contrast to the 2 % Fe doped films where the small high frequency arc is caused by the much higher ionic conductivity therein.

SI 4 XRD of Sr overstoichiometric targets

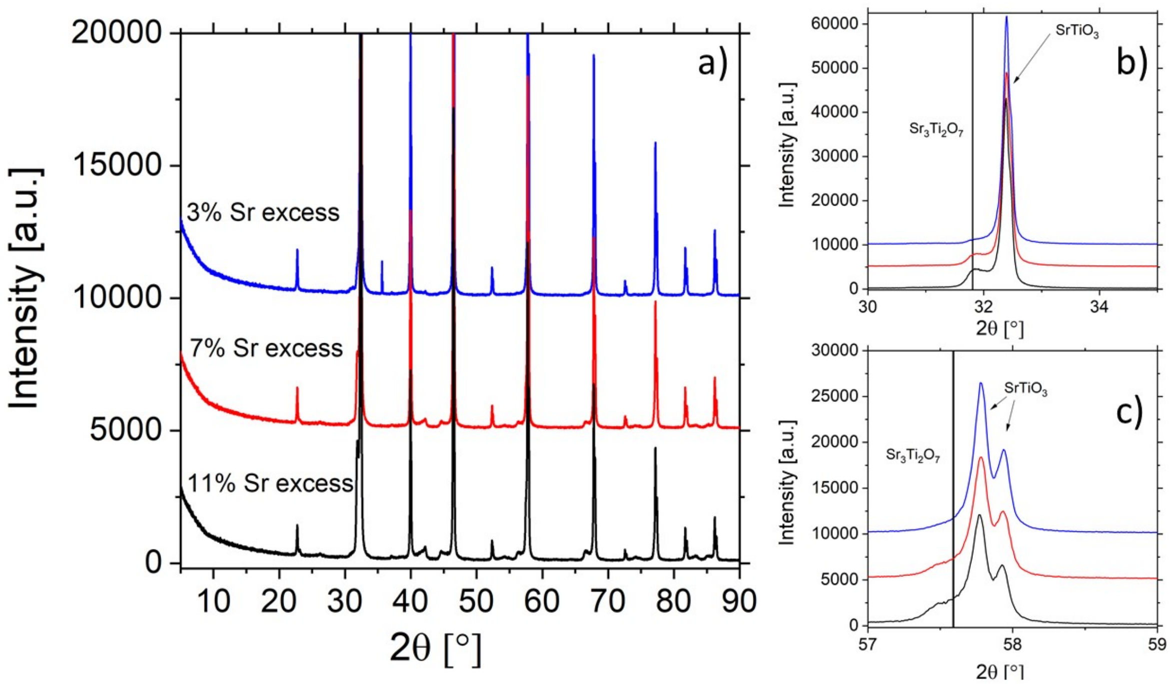

Fig. SI 2: X-ray diffraction measurements performed for polycrystalline targets of 2 % Fe-doped $SrTiO_3$ with 3 %, 7 % and 11 % Sr excess (a). The pellets consisted mainly of $SrTiO_3$ but also showed $Sr_3Ti_2O_7$ as additional phase. Not surprisingly, the amount of the secondary Sr rich phase directly correlated with the amount of excess Sr in the pellet. This can be seen in (b) and (c) of this figure. The highest Sr excess gives the highest amount of secondary phase after the described temperature treatment.

SI 5 XRD of thin films deposited from overstoichiometric targets

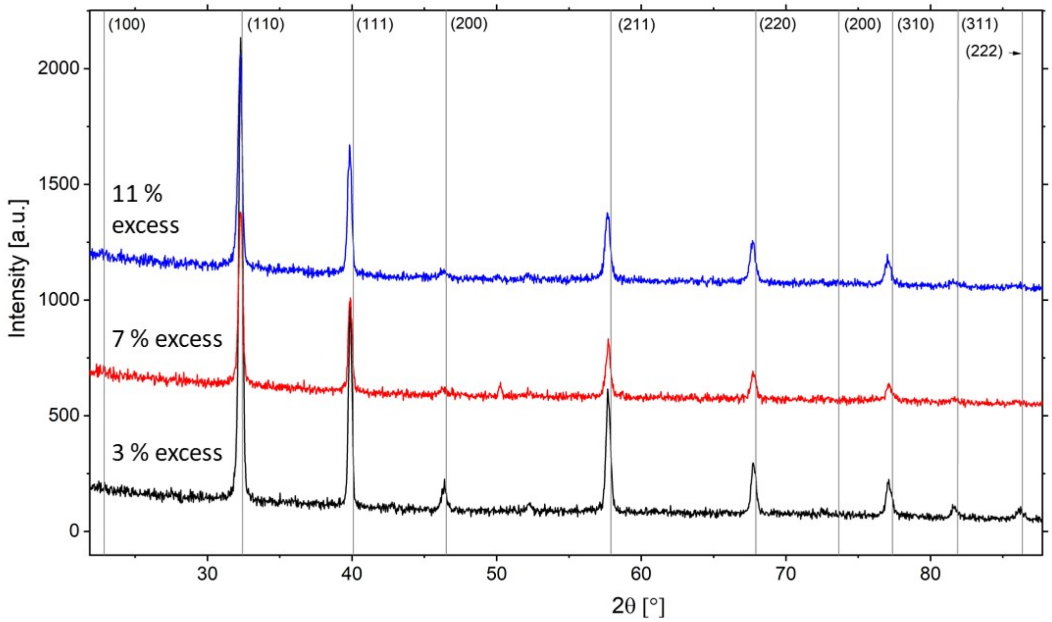

Fig. SI 3: XRD results for Fe-doped $SrTiO_3$ thin films on MgO deposited from 2 % Fe doped targets with Sr overstoichiometry of 3 %, 7 %, or 11 %, respectively. The obtained thin films are phase pure according to XRD as only $SrTiO_3$ reflexes are visible.

SI 6 Discussion of the capacitances found for the thin films deposited from Sr overstoichiometric targets

As mentioned in the main paper, the high frequency arc in the impedance spectra found for thin films deposited from Sr overstoichiometric targets is attributed to the $SrTiO_3$ bulk due to the match with expected geometrical capacitances. The capacitances of the two other R-CPE circuits fit pretty well to space charge layers; for bulk $SrTiO_3$ with 2 % Fe, a relative permittivity of 150 and a space charge potential of 600 mV, for example, ca. 6 nm result for one space charge [6]. It is thus reasonable to assume that the corresponding capacitances are due to the space charge layers at the two electrodes, with the Nb:$SrTiO_3$/Fe:$SrTiO_3$ junction probably having the smaller capacitance (due to two similarly thick space charge zones in both $SrTiO_3$ parts). Hence, the large low frequency arc would be the space charge between $SrTiO_3$ film and Pt. From the measured peak frequencies, we can estimate the space charge potential and a rather realistic value of ca. 700 mV is found [6]. Accordingly, the dc resistance does no longer reflect the electronic conductivity.

SI 7 Characterization of samples using optical microscopy after electrical measurements

From previous studies, we have atomic force microscopy (AFM) images and transmission electron microscopy (TEM) images available [2]. Using optical microscopy, we checked each sample after electrical characterization to make sure no changes in surface structure or crack formation happened during impedance measurement. Fig. SI 4 shows microscopy images of a typical sample after impedance measurements. Damages to the microelectrode after removing the Pt needle are indicated with arrows. Furthermore, we also show microelectrodes before and after measurements for thin films deposited from targets with 0 %, 5 %, 7 % and 11 % Sr excess in Fig. SI 5.

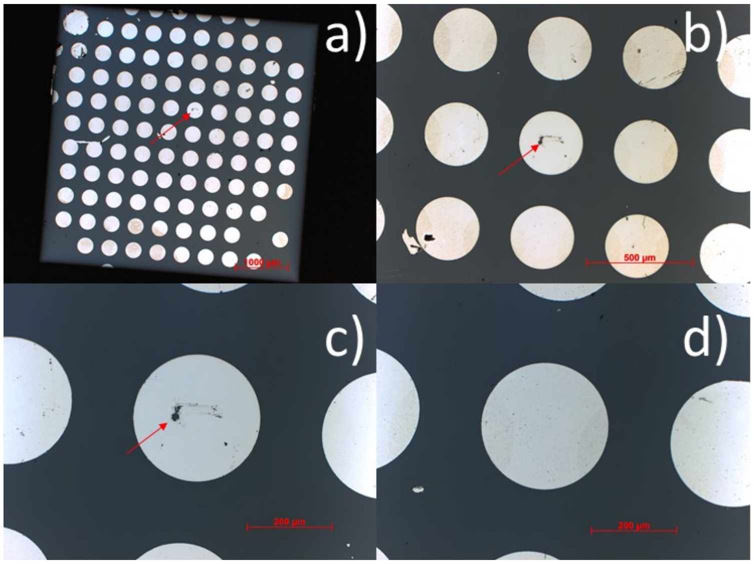

Fig SI 4: Optical microscopy images of a typical sample after impedance measurements. No changes in the surface or cracks are visible. The point where the Pt needle contacted the microelectrode is highlighted with an arrow (a-c). When removing the Pt needle, a part of the Pt electrode is usually removed as well. In (d), a microelectrode which was not contacted is shown as a reference.

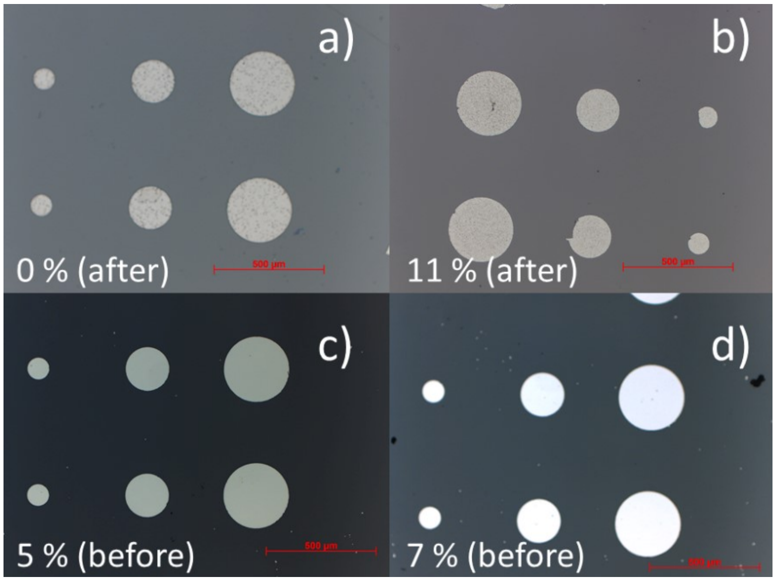

Fig. SI 5: Optical microscopy images for different samples before and after impedance measurements. Images for thin films deposited from a stoichiometric target (a) and a target with 11 % (b) Sr excess are shown for the state after measurements. Images for thin films deposited from targets with 5 % (c) and 7 % (d) Sr excess are shown before impedance measurements.

SI 8 Limitation and validity of cross-comparison for different substrate materials

Different substrates were used in this study, ranging from Nb:$SrTiO_3$ (enabling epitaxial growth of Fe:$SrTiO_3$ thin films) to MgO (yielding polycrystalline thin films). Of course, this change in substrate might also be accompanied by changes is microstructure, stress/strain, and possibly stoichiometry, which could limit cross-comparison of the results obtained for different substrates. Here, we want to address this point and show why we consider comparison of these data to be feasible.

Starting with Nb:$SrTiO_3$ and Fe:$SrTiO_3$, they differ only slightly in lattice parameter according to literature[7, 8], still enabling epitaxial growth of Fe:$SrTiO_3$ on Nb:$SrTiO_3$. These substrates were used for electrical characterization, RSM imaging and PALS measurements. Therefore, we gain information on electrical properties (EIS), structure (RSM) and predominant defect species and defect chemistry (PALS). So just from these three types of measurements on the same substrate (i.e. allowing comparison without any influence of substrate changes), we can already link the change in electrical conductivity (from pseudo-intrinsic to bulk like) with changes in the structure (from cation vacancy related deviations between the thin film and the substrate to great agreement in lattice parameters) and the vacancy type (from Sr vacancies to Ti vacancies). The vacancy type gives us important information on the nonstoichiometry accommodation. Already here it is natural to expect Sr deficiency in our pseudo-intrinsic films as we already know that we have a large amount of Sr vacancies in these films (RSM and PALS information). In contrast, there is no hint for cation vacancies in the structure of our bulk-like films. The change in predominant charge carrier further supports this point.

For chemical analysis, a change in substrate is necessary. Here, limitations arise from the fact that no Sr, Ti or Fe are allowed for a valid analysis in the substrate, not even as a trace impurity. Furthermore, no elements that interfere in OES or MS measurements must be in the substrate as well. Here, we chose MgO. While the sticking coefficient of MgO compared to Nb:$SrTiO_3$ and stress/strain in the thin films deposited on these substrate might be different, the information obtained from this system is well in line with the measurements obtained for Nb:$SrTiO_3$ substrates. A change in stoichiometry is also found, yielding ideal stoichiometry on MgO substrates for the same thin films exhibiting bulk-like conductivity on Nb:$SrTiO_3$ substrate. Hence, the general trend supports the validity of cross-comparison. The exact composition is expected to vary for different PLD setups and parameters, hence the exact amount of Sr compensation needed (here 7%) for optimized stoichiometry may also change for different PLD setups.

The PALS measurement giving information on the predominant defect helps to bridge the gap between the two different substrates. The conductivity is a result of defect chemistry, and the defect chemistry itself is a result of nonstoichiometry accommodation, thus composition of the films.

SI 9 Error of impedance measurements and fit and the impact on the conductivity

Here, we want to address the point of the validity of impedance measurements and fits since the resistance values of the fits are also relevant for the calculated conductivity. In Fig. SI 9.a, all spectra of the pseudo-intrinsic standard film measured at 551 °C are shown and in Fig. SI 9.b the corresponding error bars are shown, and for better clarity, in Fig. SI 9.c the measurement points are scaled to a smaller size. One can clearly see that the error bars are smaller than the size of the measurement points. Hence, the individual impedance measurements are in great agreement, showing high quality of the measurements.

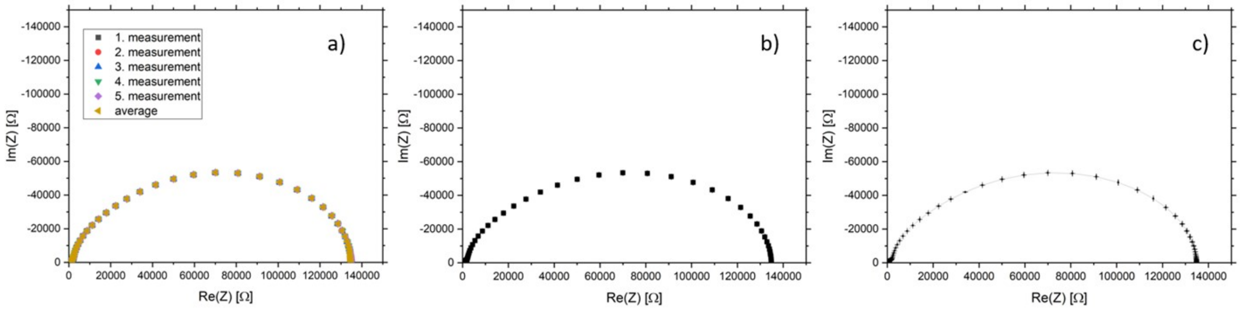

Fig. SI 6: Assessment of the quality of the impedance measurements. All individual measurements show next to no deviation (a), the error bars are smaller than symbols used (b) and (c).

To provide a measure for the quality of the fit, Tab. SI 3 also shows the absolute values, errors and relative errors of all individual fitting parameters at 551 °C (see Fig. SI 9) as well as the deviation of the individual measurements. Furthermore, the range of errors for all temperatures is

also given. Overall, errors in the low percentage range are obtained (only C_{chem} shows higher errors).

Tab. SI 3: Parameters and their respective fit values, measurement deviation, error and relative error at 551 °C as well as for the total temperature range (showing the highest and lowest values for each segment) are shown for pseudo-intrinsic standard films.

**Parameter**	**At 551 °C**			**Total temperature range**		
	**Value (fit) ± standard deviation [unit]**	**Error of the fit [unit]**	**Error % (fit) [%]**	**Value range (fit) [unit]**	**Error range (fit) [unit]**	**Error % range (fit) [%]**
$\mathbf{R_{eon}}$	$1.63*10^{5} \pm 240$	667	0.41	$1.59*10^{6}$ – 12023	8580 - 25	0.7 – 0.2
$\mathbf{R_{ion}}$	1968 ± 4	27	1.3	13998 – 310	169 - 10	3.1 – 1.2
$\mathbf{C_{chem}}$	$9.86*10^{-10} \pm 3.8*10^{-12}$	$4.7*10^{-10}$	47	$9.8*10^{-10}$ – $1.5*10^{-9}$	$5.9*10^{-10}$ – $4.6*10^{-10}$	47 – 34
**CPE-T$_{cont}$**	$2.35*10^{-8} \pm 3*10^{-11}$	$5.8*10^{-10}$	2.5	$2.34*10^{-8}$ – $2.5*10^{-8}$	$5.8*10^{-10}$ – $6.8*10^{-10}$	2.7 – 2.5
**CPE-P$_{cont}$**	$0.82 \pm 1.2*10^{-2}$	$2.5*10^{-3}$	0.3	0.82 – 0.84	$2.5*10^{-3}$- $3.2*10^{-3}$	0.4 – 0.3
$\mathbf{C_{geom}}$	$2.02*10^{-10} \pm 2.60*10^{-14}$	$2.2*10^{-12}$	1.0	$2.2*10^{-10}$ – $1.7*10^{-10}$	$1*10^{-12}$ – $9*10^{-12}$	5 – 0.5

As a comparison, the corresponding plot and table for the thin film deposited from a target with 7 % Sr overstoichiometry yielding bulk-like conductivity are shown in Fig. SI 10 and Tab. SI 4.

The impedance spectra were measured at 327 °C. Fig. SI 10 shows the quality of the impedance measurements for the thin films deposited from bulk-like targets.

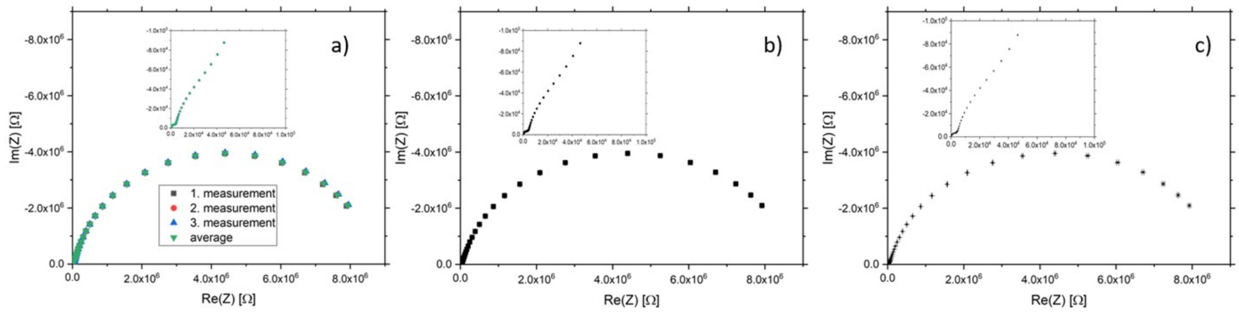

Fig. SI 7: Assessment of the quality of the impedance measurements. All individual measurements are plotted in (a) and they show great agreement. The error bars are smaller than the measurement points in (b). A downsizing of the measurement points reveals the error bars (c), proving that the errors are indeed very small.

Tab. SI 4 shows the error of the fit. Again, errors in the low percentage range are obtained, showing that fits with high quality were obtained.

The impact of the errors in the fit are then shown in Fig. SI 11 for the respective conductivity values in Arrhenius plots. Considering that the samples differ by 5 orders of magnitude, an error in the low percentage range is barely visible in an Arrhenius plot. Magnifications (see Fig. SI 11.b and c) show the upper and lower limits of the 95 % confidence interval.

Tab. SI 4: *Parameters and their respective fit values, measurement deviation, absolut error and relative error at 327 °C as well as for the total temperature range (showing the highest and lowest values for each segment) are shown for films deposited from targets with 7 % Sr overstoichiometry*

**Parameter**	**At 327 °C**			**Total temperature range**		
	**Value (fit) ± deviation [unit]**	**Error (fit) [unit]**	**Error % (fit) [%]**	**Value range (fit) [unit]**	**Error range (fit) [unit]**	**Error % range (fit) [%]**
**R1**	4025 ± 20	36	0.9	1102 – 43198	11 – 292	0.7 – 1.0
**CPE-T1**	$2.1*10^{-10} \pm 4*10^{-14}$	$1.3*10^{-12}$	0.6	$1.9*10^{-10}$ – $2.3*10^{-10}$	$1.4*10^{-12}$ – $9.5*10^{-13}$	0.4 – 0.9
**CPE-P1**	1 (fixed)	- (fixed)	-	1 (fixed)	- (fixed)	-
**R2**	52994 ± 274	1382	2.6	13557 – $4.4*10^{5}$	384 – 12637	2.3 – 2.9
**CPE-T2**	$5.6*10^{-9} \pm 1*10^{-11}$	$3.2 *10^{-10}$	5.7	$6*10^{-9}$ – $3*10^{-9}$	$4.8*10^{-10}$ – $1.4*10^{-10}$	4 – 7
**CPE-P2**	$0.90 \pm 2*10^{-4}$	$5*10^{-3}$	0.6	0.90 – 0.95	$5*10^{-3}$ – $7*10^{-3}$	0.5 – 0.7
**R3**	$8.9*10^{6}$ ± 46889	45978	0.5	$2.5*10^{8}$ – $1.4*10^{6}$	$1.4*10^{7}$ – 4864	0.3 – 5.7
**CPE-T3**	$5.6*10^{-9} \pm 4*10^{-12}$	$5.3*10^{-11}$	1.0	$5.0*10^{-9}$ – $6.2*10^{-9}$	$3.6*10^{-11}$ – $8.0*10^{-11}$	0.7 – 1.3
**CPE-P3**	$0.91 \pm 1*10^{-4}$	$2*10^{-3}$	0.2	0.90 – 0.91	$0.2*10^{-3}$	0.2

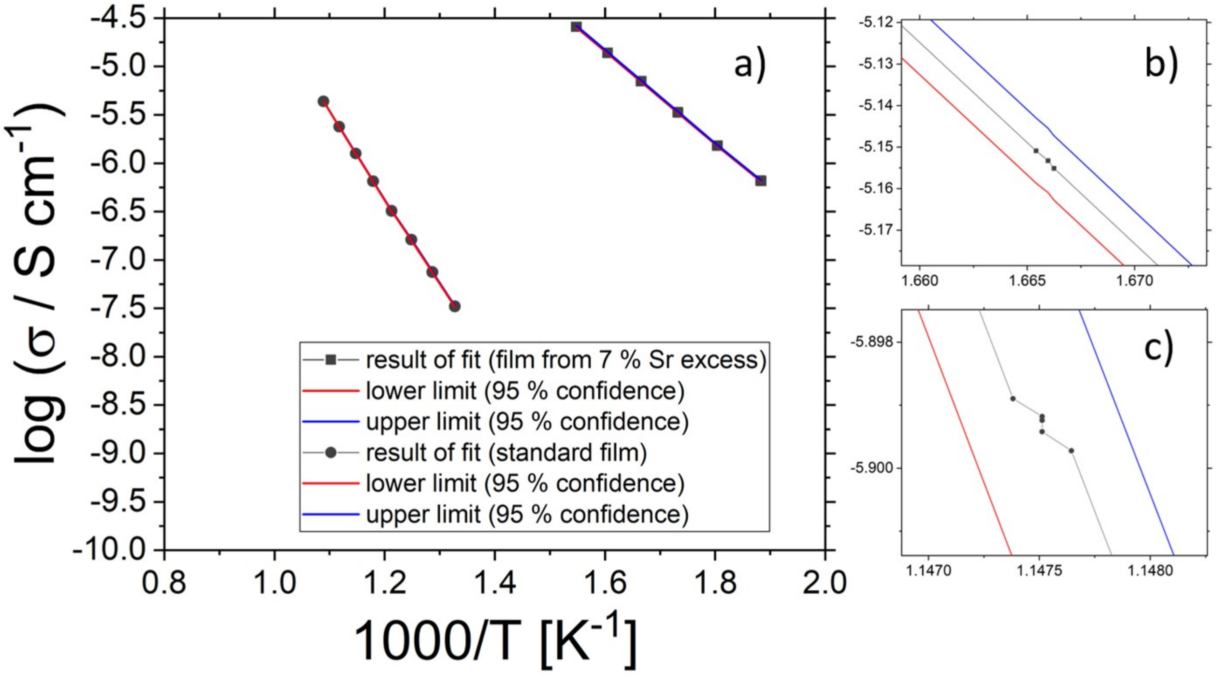

Fig. SI 8: Confidence intervals for the Arrhenius plots of the standard sample and the thin film deposited from a target with 7 % Sr excess (a). The upper and lower limits of the 95 % intervals are given in blue and red, respectively. Magnifications are shown for the thin film deposited from a target with 7 % Sr excess (b) as well as the pseudo-intrinsic thin film deposited from a stoichiometric target (c).

References:

1. De Souza, R.A., J. Fleig, J. Maier, O. Kienzle, Z. Zhang, W. Sigle, and M. Rühle, *Electrical and structural characterization of a low-angle tilt grain boundary in iron-doped strontium titanate.* Journal of the American Ceramic Society, 2003. **86**(6): p. 922-928.
2. Kubicek, M., S. Taibl, E. Navickas, H. Hutter, G. Fafilek, and J. Fleig, *Resistive states in strontium titanate thin films: Bias effects and mechanisms at high and low temperature.* Journal of Electroceramics, 2017. **39**(1-4): p. 197-209.
3. Taibl, S., G. Fafilek, and J. Fleig, *Impedance spectra of Fe-doped* $SrTiO_3$ *thin films upon bias voltage: Inductive loops as a trace of ion motion.* Nanoscale, 2016. **8**(29): p. 13954-13966.
4. Kubicek, M., A. Limbeck, T. Frmling, H. Hutter, and J. Fleig, *Relationship between cation segregation and the electrochemical oxygen reduction kinetics of* $La_{0.6}Sr_{0.4}CoO_{3-\delta}$ *thin film electrodes.* Journal of the Electrochemical Society, 2011. **158**(6): p. B727-B734.
5. Denk, I., W. Münch, and J. Maier, *Partial Conductivities in* $SrTiO_3$*: Bulk Polarization Experiments, Oxygen Concentration Cell Measurements, and Defect-Chemical Modeling.* Journal of the American Ceramic Society, 1995. **78**(12): p. 3265-3272.
6. Ohly, C., S. Hoffmann-Eifert, X. Guo, J. Schubert, and R. Waser, *Electrical Conductivity of Epitaxial* $SrTiO_3$ *Thin Films as a Function of Oxygen Partial Pressure and Temperature.* Journal of the American Ceramic Society, 2006. **89**(9): p. 2845-2852.
7. Lenser, C., A. Koehl, I. Slipukhina, H. Du, M. Patt, V. Feyer, C.M. Schneider, M. Lezaic, R. Waser, and R. Dittmann, *Formation and Movement of Cationic Defects During Forming and Resistive Switching in* $SrTiO_3$ *Thin Film Devices.* Advanced Functional Materials, 2015. **25**(40): p. 6360-6368.
8. Ohnishi, T., K. Shibuya, T. Yamamoto, and M. Lippmaa, *Defects and transport in complex oxide thin films.* Journal of Applied Physics, 2008. **103**(10).
